# Supplementary material for: Optimization of Infrared Heating Conditions for Precooked Cowpea Production Using Response Surface Methodology
Source: Molecules. 2021 Oct 11;26(20):6137. doi: 10.3390/molecules26206137 (PMC8541054; doi:10.3390/molecules26206137)
Supplement: Supplementary file 1 [file molecules-26-06137-s001.zip › molecules-1394215-supplementary.pdf]

Supplementary Figure

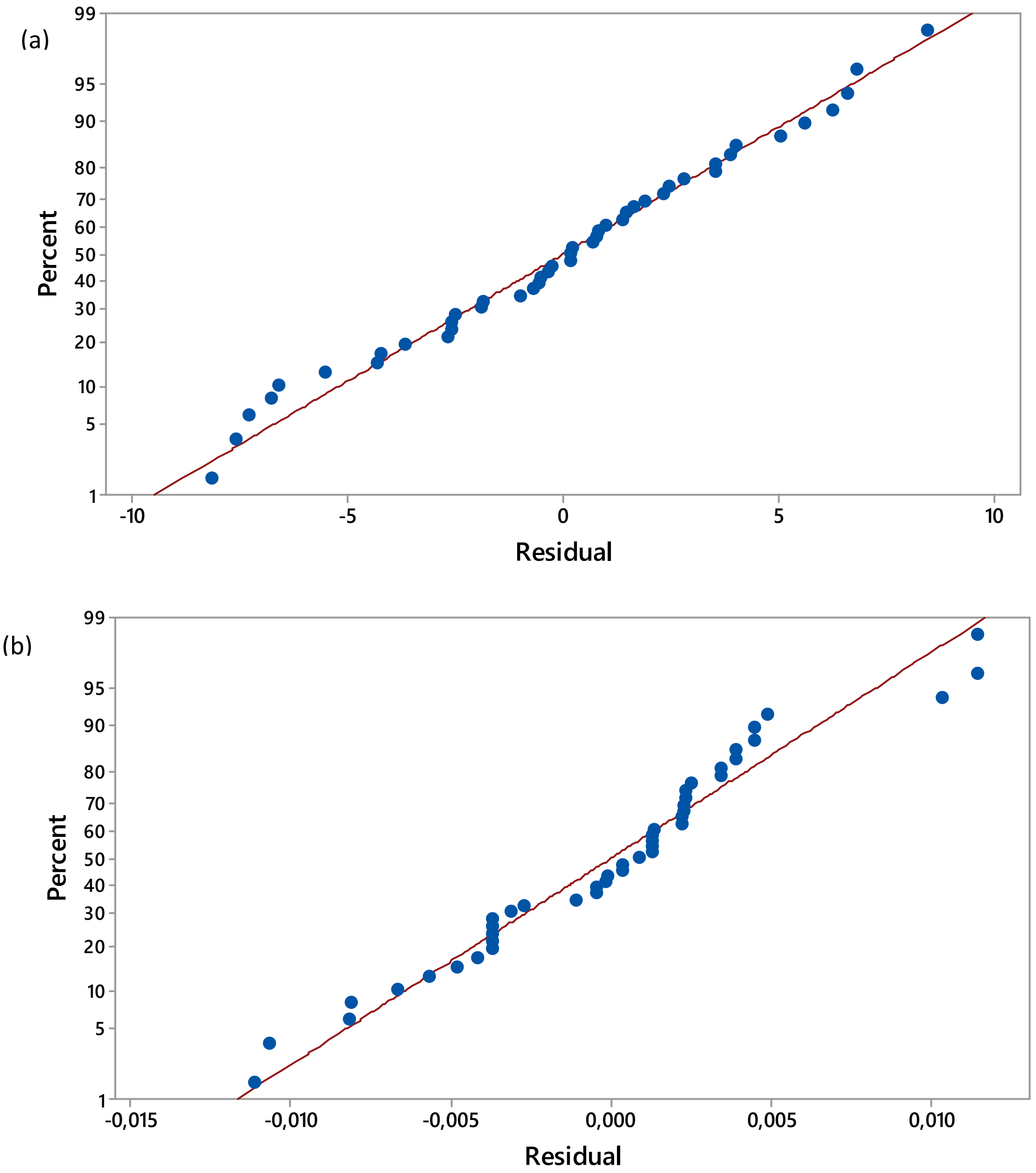

Figure S1: Normal distribution of residuals (a) Water absorption capacity and (b) Bulk density.
